# Time Is Key: Early Diagnosis of Post-Transplant Lymphoproliferative Disorder Presenting as Primary CNS Diffuse Large B-Cell Lymphoma

**DOI:** 10.3390/curroncol33060333

**Published:** 2026-06-04

**Authors:** Asli Altunbas, Aarti Desai, Andrea Muniz, Hussien Al Asi, Rajvi Chaudhary, Laxmi Raj Bangari, Surbhi Dadwal, Jose Ruiz, Juan Leoni, Julie Hammack, Harry Powers, James Foran, Rohan Goswami

**Affiliations:** 1 Division of Heart Failure and Transplantation, Mayo Clinic, Jacksonville, FL 32224, USA; sabireasli.altunbas@bahcesehir.edu.tr (A.A.); desai.aarti@mayo.edu (A.D.); muniz.andrea@mayo.edu (A.M.);; 2 Computational Pathology and AI, Department of Laboratory Medicine and Pathology, Mayo Clinic, Jacksonville, FL 32224, USA; 3 Department of Neurology, Mayo Clinic, Jacksonville, FL 32224, USA; 4 Division of Infectious Diseases, Mayo Clinic, Jacksonville, FL 32224, USA; powers.harry@mayo.edu; 5 Division of Hematology, Mayo Clinic, Jacksonville, FL 32224, USA

**Keywords:** post-transplant lymphoproliferative disorder, primary CNS lymphoma, heart transplant, Epstein–Barr virus, immunosuppression, intracerebral hemorrhage

## Abstract

Post-transplant lymphoproliferative disorder (PTLD) involving the central nervous system (CNS) is a rare but serious life-threatening complication seen in recipients of solid organ transplant. Primary CNS encompasses 5–15% of all types of PTLD diagnoses, and heart transplant recipients represent 3–5% of those reported cases. Diagnosis is often delayed due to the highly variable presentation, with some cases remaining undiagnosed for years. Multidisciplinary collaboration is crucial for early diagnosis and management. A 53-year-old woman patient presented with altered mental status. MRI revealed nodular ventriculitis and bilateral periventricular hyperdense infiltrates. CSF studies demonstrated lymphocytic pleocytosis, elevated protein, and EBV-PCR-positive results. A stereotactic brain needle biopsy confirmed the presence of EBV-positive diffuse large B-cell lymphoma, consistent with primary CNS PTLD, 14 months after her heart transplant. Despite appropriate management, the patient experienced progressive neurological decline and ultimately suffered a fatal intracerebral hemorrhage. We demonstrate the importance of the early diagnosis and variable presentation of post-heart-transplant PTLD. The importance of surveillance regardless of EBV status and close monitoring of disease progression due to potential life-threatening complications, such as fatal hemorrhages. Therefore, primary CNS-PTLD remains a challenging disease and is being increasingly recognized with improved transplant recipient survival and prolonged exposure to chronic immunosuppression.

## Simple Summary

Primary central nervous system post-transplant lymphoproliferative disorder is an uncommon but potentially fatal complication after organ transplantation. Because its symptoms can be vague and overlap with those of infection, medication toxicity, or other brain lesions, diagnosis may be delayed. In this report, we describe a heart transplant recipient who developed confusion 14 months after transplantation while receiving immunosuppressive therapy. Brain imaging, cerebrospinal fluid testing, and ultimately brain biopsy led to the diagnosis of EBV-positive diffuse large B-cell lymphoma involving the central nervous system. Her course was complicated by progressive neurological decline and intracerebral hemorrhage despite multidisciplinary care. This case emphasizes that clinicians should maintain suspicion for CNS-PTLD in recipients of transplants with new neurological symptoms, even when presentation occurs relatively early after transplant or when risk factors appear limited. Early imaging, EBV testing, tissue diagnosis, and close follow-up are important because the disease can progress rapidly and may lead to life-threatening complications.

## 1. Introduction

### 1.1. Epidemiology of Post-Transplant Malignancies

De novo malignancies represent significant long-term complications following solid organ transplants (SOTs). In a study involving 51,597 adult recipients of heart transplants with a 20-year follow-up, up to one in four cases of post-SOT de novo malignancies were identified [1]. Among these, post-transplant lymphoproliferative disorder (PTLD) was the second most common malignancy seen, with a 1–10% annual incidence of all SOTs [2], second to non-melanoma skin cancers [3]. Development of PTLD is most commonly seen after intestinal and multi-organ transplants, with an incidence of 5–20%, followed by lung and heart transplantation at 2–10%, and, lastly, liver transplant at 1–5% [4]. Primary central nervous system PTLD (PCNS-PTLD) is seen in only 5–15% of all PTLD cases, most of which are seen in recipients of renal transplant (46–74%), followed by hematopoietic stem cell transplant (27%), liver transplant (11%), and finally heart transplant (3–5%) [5].

### 1.2. Pathophysiology of Primary CNS-PTLD

In immunocompetent individuals, primary EBV infection is kept dormant in memory B cells without triggering active viral replication [4]. However, the chronic immunosuppression required in patients after transplant to prevent allograft rejection creates a favorable environment for uncontrolled EBV proliferation and the development of lymphoma [4]. EBV seropositive status plays a central role in PTLD, accounting for up to 90% of reported cases [3]. The occurrence of PTLD is strongly associated with the dose and duration of immunosuppression and pretransplant EBV IgG status. Most of these cases are EBV-related monomorphic diffuse large B-cell lymphomas (DLBCLs) [6]. Despite expressing an immunogenic virus, EBV-associated PCNSL in patients who are immunosuppressed is immunobiologically different from EBV-negative HIV-negative PCNSL, and these patients continue to exhibit EBV antigens [7]. EBV-negative PCNSL demonstrates enrichment in the activated B-cell (ABC) cell-of-origin subtype and frequent MYD88, CD79B, and PIM1 mutations. On the other hand, the ABC frequency was low, and these mutations were not present in any individuals who were EBV-tissue-positive [7]. Additionally maintained antigen presentation is suggested by rarity of copy number loss in HLA class I/II and antigen-presenting/processing genes [7]. Despite this retained antigen presentation profile, EBV-positive PCNSL demonstrates the features of a tolerogenic tumor microenvironment, including increased macrophage and immune-checkpoint-related gene expression [7,8]. Further transcriptomic and epigenetic profiling supports EBV-positive PCNSL as a distinct biological subgroup, with EBV-associated gene expression programs and the upregulation of interleukin-10/JAK-STAT, NOTCH, and viral life-cycle pathways, whereas EBV-negative tumors show stronger B-cell receptor and WNT/β-catenin signaling [9].

Isolated CNS involvement is rare, and its nonspecific clinical presentation makes the diagnosis of PTLD particularly challenging [3]. Primary CNS-PTLD is often characterized by atypical symptoms such as headache, mental status changes, epileptic seizures, and weakness of motor function [10]. Imaging can also present a challenge, as many intracranial pathologies, including primary CNS lymphoma, glial tumors, metastatic disease, and intracranial abscesses, can share similar radiographic characteristics [5]. The median time for diagnosis after the transplant is 54 months [11]. Diagnostic delays could also be attributed to the overlapping symptoms with other post-transplant complications, including opportunistic infections and medication toxicity [3]. This insidious onset and subtle early manifestations lead to misdiagnosis or delayed presentation, requiring high clinical suspicion in patients post-transplant with neurological symptoms.

We present a case of a 53-year-old female with a history of nonischemic dilated cardiomyopathy who underwent heart transplant and was subsequently diagnosed with PCNS-PTLD 14 months post-heart transplant.

## 2. Case Presentation

A 53-year-old female with a history of nonischemic dilated cardiomyopathy status 14 months post-transplant presented with confusion. Her past medical history was significant for hypertension, hypercholesterolemia, hypothyroidism, and a coccidiomycosis-positive pulmonary nodule found incidentally during pre-transplant evaluation. She was transferred to our facility after evaluation at an outside facility due to altered mental status, and initial investigations showed a possible frontoparietal mass. Her outpatient medications included mycophenolate mofetil 750 mg BID, tacrolimus 1 mg in the morning and 0.5 mg in the evening, fluconazole 400 mg OD, ezetimibe 10 mg OD, atorvastatin 80 mg OD, aspirin 81 mg OD, and levothyroxine 75 mcg OD.

The heart transplant team was consulted immediately for immunosuppression management, which was changed to mycophenolate mofetil 750 mg IV BID and tacrolimus 0.5 mg SL BID to ensure compliance owing to altered mental status. No evidence of graft dysfunction or rejection was detected since the transplantation. The last echocardiogram during follow-up showed normal left and right ventricular size and function, with a left ventricular ejection fraction of 67% and no significant valvular disease.

Infectious diseases and neurology were consulted after an initial MRI brain scan with and without IV contrast was found to be concerning for nodular ventriculitis secondary to atypical or opportunistic infection or mass lesion. There was persistent vasogenic edema surrounding the enhanced lesions, with the unchanged mass effect resulting in the compression of the lateral ventricles, without midline shift or hydrocephalus (Figure 1).

Based on the axial MRI findings, the leading consideration was a lymphoproliferative process involving the CNS, particularly primary CNS-PTLD, while atypical infection was considered less likely.

Head CT was performed at the request of neurology to help further the diagnostic differential and revealed bilateral hyperdense periventricular/subependymal infiltrates with adjacent vasogenic edema, worse along the left lateral ventricle, raising suspicion of primary CNS-PTLD (Figure 2). Further systemic imaging including a contrast-enhanced CT of the chest, abdomen and pelvis was performed, and it revealed no evidence of systemic lymphoproliferative disease (Figure 3). While PCNS-PTLD is not formally staged, the Lugano classification indicated Stage IV due to the extensive extranodal involvement without a primary nodal focus [12].

Lumbar puncture (LP) showed nucleated cells 79/mcL (reference range 0–5/mcL), lymphocytes 93%, proteins 202 mg/dL (reference range 15–45 mg/dL), and positivity for EBV PCR, further guiding the investigation of PTLD. She was treated with levetiracetam 1000 mg PO BID for seizure prophylaxis. Due to diagnostic uncertainty and immunocompromised state, she was started on empiric antimicrobials with imipenem-cilastatin 500 mg IV QID, trimethoprim–sulfamethoxazole 5 mg/kg IV TID, amphotericin B 5 mg/kg IV OD, caspofungin 150 mg IV OD, and acyclovir 10 mg/kg TID until biopsy confirmation.

Stereotactic brain needle biopsy was performed on the right frontal brain mass and showed viable and necrotic areas of specimen with multifocal angiocentrically arranged clusters of atypical mononuclear cells exhibiting mitotic activity, confirming EBV-positive diffuse large B-cell lymphoma consistent with primary CNS-PTLD (Figure 4). All molecular and cytogenetic studies were performed using tissue samples from the brain. Cerebrospinal fluid studies were not conducted in lieu of the brain biopsy. Histopathologic examination of the brain tissue demonstrated viable and necrotic fragments involved by multifocal, angiocentrically arranged clusters of atypical mononuclear cells with mitotic activity (Figure 4A–C). Immunohistochemical staining performed on block B1 showed that the atypical cells were strongly positive for CD20, BCL-2, and MUM-1, supporting B-cell lineage with an activated B-cell phenotype. The cells were negative for CD3, CD10, BCL-6, and C-MYC. MIB-1/Ki-67 demonstrated a proliferative index of approximately 50%. EBV in situ hybridization was positive (Figure 4D), with RNA EBV-ISH showing stronger and more diffuse positivity (Figure 4E). Fluorescence in situ hybridization studies did not detect MYC rearrangement, MYC-IGH fusion, BCL2 rearrangement, or BCL6 rearrangement. Overall, these findings were consistent with an EBV-positive large B-cell lymphoproliferative process involving the central nervous system.

Her empirical treatment for aseptic meningitis (imipenem–cilastatin, trimethoprim sulfamethoxazole, amphotericin B, and caspofungin) was discontinued, and she was started on dexamethasone 4 mg PO TID, which improved her alertness and cognition. Baseline CBC prior to chemotherapy showed leukocytes 8.0 × 10^9^/L (reference range 3.4–9.6 × 10^9^/L), platelets 234 × 10^9^/L (157–371 × 10^9^/L), and erythrocytes 3.32 × 10^12^/L (3.92–5.13 × 10^12^/L). Following consultation with hematology and oncology, rituximab 375 mg/m^2^ was initiated once weekly for 2 months. Cytarabine administration was delayed due to worsening renal function on day 9 of hospitalization (eGFR 19 mL/min/m^2^). Her immunosuppression regimen was continued with tacrolimus 3 mg daily, and mycophenolate mofetil was lowered to 250 mg BID due to PTLD. The tacrolimus trough level was between 4 and 6 ng/mL due to her PTLD. Her renal function improved over the following week (eGFR 44 mL/min/m^2^), allowing for initiation of cytarabine 1 g IV BID for four consecutive days.

Given multiple risk factors for delirium, precautions such as maintaining a healthy sleep/wake cycle, guided cognitive and environmental stimulation, and reorientation were recommended. Unfortunately, the patient developed delirium secondary to acute metabolic encephalopathy, most likely due to high-dose IV steroids and rituximab. Her waxing and waning mentation continued; she developed abulia and akinetic mutism. Physical, speech, and occupational therapy evaluations were conducted, and a multidisciplinary team agreed that the patient was suitable for discharge to an inpatient rehabilitation center. She developed acute kidney injury, which resolved prior to discharge. She was also noted to have steroid-related hypertension managed with amlodipine 5 mg PO OD. Prior at-home medications were continued with the addition of levetiracetam 1 gm PO BID for anti-seizure prophylaxis and dexamethasone 4 mg PO OD for cerebral edema. Aspirin was discontinued due to thrombocytopenia and the high risk of bleeding due to chemotherapy. The discharge plan included complete blood count and comprehensive metabolic panel testing twice weekly as well as transfusions as required for Hb < 7 g/dL or platelet count < 10,000/µL.

Approximately a month after discharge, while receiving care at a rehabilitation facility, she returned to the emergency department due to progressive physical and cognitive decline, becoming nonverbal with minimal movement of the lower extremities. She was readmitted for further investigation and her second cycle of chemotherapy. Follow-up brain CT imaging demonstrated a lesion raising suspicion of intracerebral hemorrhage without extension into the ventricles. Neurological examination revealed findings consistent with encephalopathy in the setting of poor cognitive reserve. The patient again exhibited akinetic mutism and abulia, with a paucity of spontaneous movement and resistance to passive movement, more pronounced in the upper extremities.

Given her poor prognosis and lack of clinical improvement, an extensive goals-of-care discussion was held with the multidisciplinary team and the patient’s family. The decision was made to transition to hospice care. Following admission to hospice care, the patient’s spouse elected to resume home management and requested to reinitiate immunosuppressive therapy with tacrolimus 2 mg BID and mycophenolate mofetil (Cellcept) 250 mg BID. After 20 days in hospice, the patient presented to the emergency department with declining mental status and pain and was admitted for comfort measures only. Despite supportive care, the patient’s condition continued to deteriorate, and she passed away two weeks later.

## 3. Discussion

### 3.1. Significance of Early Diagnosis

Our case underscores the importance of early recognition and interdisciplinary collaboration in diagnosing and managing PCNS-PTLD. Our patient was diagnosed 14 months post-transplant, which represents a considerably early presentation of PCNS-PTLD compared to the median of 54 months reported in an international cohort of 84 cases of PTLD [11] and 53 months in a study including 34 patients PTLD [3]. Our patient’s risk factors were minimal, with immunosuppression being the only notable factor when compared to the data for the majority of other CNS-PTLD cases found in the literature.

Despite the importance of early diagnosis, as mentioned, the nonspecific clinical presentation and overlapping radiographic findings often make early recognition challenging. The initial imaging findings in our case pose a diagnostic challenge in distinguishing PCNS-PTLD from other potential causes. The differential diagnoses in PCNS-PTLD include PCNS lymphoma, glioblastoma, cerebral abscess, and infections such as cryptococcus, aspergillosis, and tuberculosis. According to White et al., multifocal, supratentorial, and lobar, ring-enhancing lesions with ill-defined margins are typical findings of PCNS-PTLD; therefore, imaging findings should be considered in conjunction with CSF EBV PCR to support the diagnosis. However, histopathologic biopsy remains the definitive diagnostic modality for PCNS-PTLD [13]. PCNS-PTLD may present with hemorrhagic lesions. White et al. reported that 7.8% of lesions demonstrated hemorrhage, and Lake et al. reported that up to 56% of lesions were hemorrhagic. However, only a few cases were significant enough to cause acute neurological symptoms [14]. In our patient, altered mental status corresponded with imaging evidence of intracerebral hemorrhage, further complicating clinical assessment and emphasizing the need for timely recognition.

### 3.2. Clinical Course and Therapeutic Challenges

Patients with PTLD are closely monitored with repeated clinical assessment, EBV-PCR in cases with high viral load, and follow-up imaging. Management typically involves reducing immunosuppression to 25–50% of the initial dose or completely stopping it if the disease is severe. Initiating anti-CD20 monoclonal antibody rituximab or other immunosuppressants such as mycophenolate or azathioprine as early as possible is key [15]. In case of multifocal CNS involvement, multidrug chemotherapy, including cyclophosphamide, vincristine, doxorubicin, and prednisolone, is added [16]. A cohort study of 84 cases of primary CNS-PTLD achieved complete resolution and prolonged progression-free survival with early detection using MRI for focal deficits, EBV PCR in blood, rapid initiation of first-line therapy with rituximab, and suspension of immunosuppressants [11].

The clinical course in this case also highlights the nuanced balance between maintaining immunosuppression to prevent graft rejection and reducing it to control lymphoproliferative disease. Reducing immunosuppression to the lowest level tolerated is the mainstay of treatment in patients diagnosed with CNS-PTLD. However, it increases the risk of rejection, and the risk–benefit ratio must be carefully discussed [5]. Decreasing the calcineurin inhibitors to at least half of the dose while discontinuing other immunosuppressives is the recommended regimen. However, this is not always possible to establish because of the risk of rejection [17]. Our heart transplant team, in collaboration with neurology and nephrology, maintained tacrolimus and mycophenolate doses of 2 mg BID and 250 mg BID, respectively.

Given these challenges, the importance of surveillance for graft dysfunction and rejection should not be underestimated. In CNS-PTLD, monomorphic PTLD accounts for 83% of cases, and reducing immunosuppressants alone has been shown to be ineffective [2]. Patients who do not respond to monotherapy are considered high risk, and an anthracycline-based chemotherapy regimen (rituximab, cyclophosphamide, doxorubicin, vincristine, prednisolone) is initiated, as this regimen is effective for long-term disease-free survival [18]. As mentioned, EBV-driven malignancies are unlikely to respond to conservative management, and outcomes with aggressive chemotherapy have generally been less favorable than in cases managed only with reduction of immunosuppression, adding to the complexity of management and prognosis [15].

CNS involvement is be a poor prognostic factor in patients with PTLD. Other poor prognostic factors include age > 55 years, elevated serum creatinine (>1.5 mg/dL), elevated LDH, ECOG status ≥ 2, and involvement of more than one site [6]. Median survival rates vary across case series, with some reporting a median survival of 13 weeks post-diagnosis [5]. According to Cavaliere et al., the median survival for CNS-PTLD is 47 months [3]. An important prognostic factor is EBV serostatus: EBV infection before transplant confers the recipient with EBV seronegative status. It is considered the highest risk of developing primary EBV CNS-PTLD infection and confers poor prognosis. Unlike our case, where donor and recipient status ere positive (donor and recipient EBV-positive), making it a rare but potentially better prognosis if diagnosed and treated promptly [19].

### 3.3. Rise in Epidemiology of PCNS-PTLD and Updates

Although still rare, both PTLD and CNS-PTLD are being diagnosed more frequently, reflecting the rising number of transplant procedures and improved survival; as a result, these disorders are becoming an increasingly important clinical concern [1,20]. We present our case to attract the attention of physicians to consider CNS-PTLD among their differential diagnoses, especially if they are dealing with post-transplant patients who have vague symptoms such as headache, mental status change, dizziness, or neurological deficits with the characteristic imaging findings suggestive of PTLD. Moreover, our case highlights the potential complication of hemorrhage within the tumor, which should be considered in patients with progressive cognitive decline and a lack of clinical improvement despite appropriate management. The importance of early diagnosis and the initiation of appropriate therapy should be appreciated, as outcomes are considerably better and mortality from the disease is significantly higher otherwise.

The recent International Primary CNS Lymphoma Collaborative Group (IPCG) study provides a useful contemporary framework for interpreting this case within the broader category of immunodeficiency-associated primary CNS lymphoma (ID-PCNSL), including post-transplant immunodeficiencies in 41.2% of their cohort (127/308) [21]. Our patient fit this phenotype, having developed EBV-positive diffuse large B-cell lymphoma confined to the CNS 14 months after heart transplantation while receiving tacrolimus and mycophenolate mofetil. Her bilateral periventricular/subependymal infiltrative lesions with nodular ventriculitis also highlight the variable imaging presentation of PCNS-PTLD in immunosuppressed hosts. The IPCG prognostic model incorporates three adverse variables: age > 60 years, Karnofsky Performance Status (KPS) < 70, and EBV positivity. In our case, the patient was 53 years old and therefore did not meet the age-related adverse criterion. However, she had EBV-positive disease and impaired functional status, most consistent with KPS < 70, meeting two IPCG adverse prognostic markers, and placing her in the intermediate-risk IPCG category. Although this category was associated with a median overall survival of approximately 29 months in the IPCG cohort, our patient experienced a fulminant clinical course, with progressive neurologic decline, intracerebral hemorrhage, transition to hospice care, and death within a short time after diagnosis. This discordance underscores that IPCG risk stratification provides useful prognostic context but may not fully capture acute neurologic complications, hemorrhagic events, or rapidly progressive CNS-PTLD in individual post-transplant patients.

## 4. Conclusions

This case report illustrates an unusually early presentation of primary CNS-PTLD in a recipient of a heart transplant with minimal risk factors. We highlight the importance of maintaining a high index of suspicion of CNS-PTLD regardless of the time elapsed since transplantation or the patient’s EBV risk status. Survival rates have improved primarily due to new targeted therapies. However, cases such as this highlight the rapidity and lethality of PTLD.

## Figures and Tables

**Figure 1:**
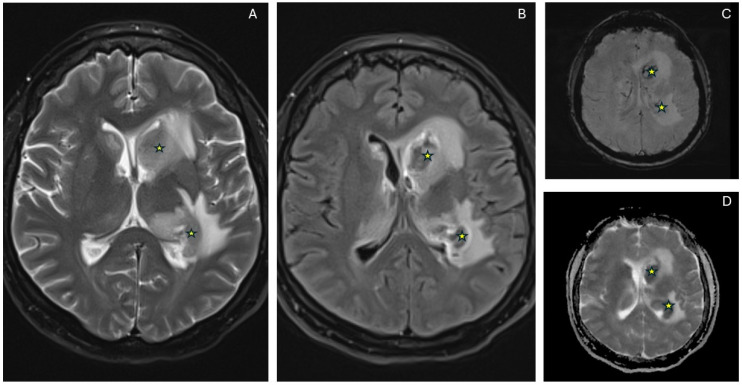
Axial brain MRI demonstrating bilateral periventricular and subependymal infiltrative lesions in post-transplant lymphoproliferative disorder (PTLD) (yellow stars). (**A**) T2-weighted and (**B**) T2-FLAIR images demonstrate hyperintense lesions along the lateral ventricles. (**C**) Susceptibility-weighted imaging (SWI) demonstrates persistent subependymal deposits involving third and fourth ventricles. (**D**) Diffusion-weighted imaging (DWI) demonstrates restricted diffusion within periventricular lesions, consistent with hypercellularity.

**Figure 2:**
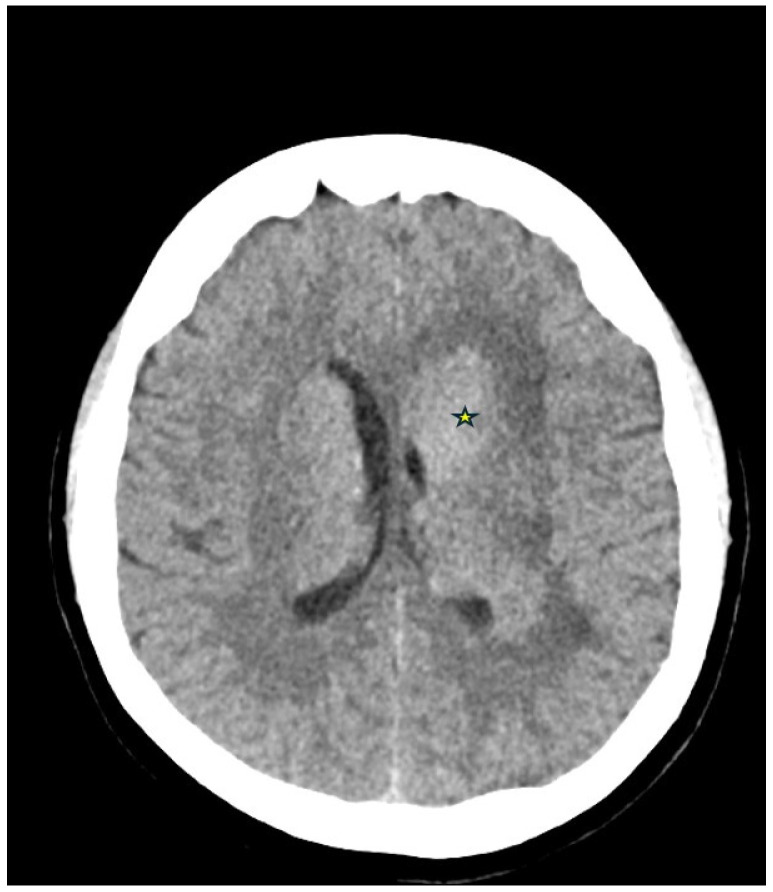
Axial non-contrast CT of the head demonstrates similar bilateral periventricular and subependymal infiltrative lesions along the lateral ventricles with a persistent mass effect (yellow star). No hydrocephalus was present.

**Figure 3:**
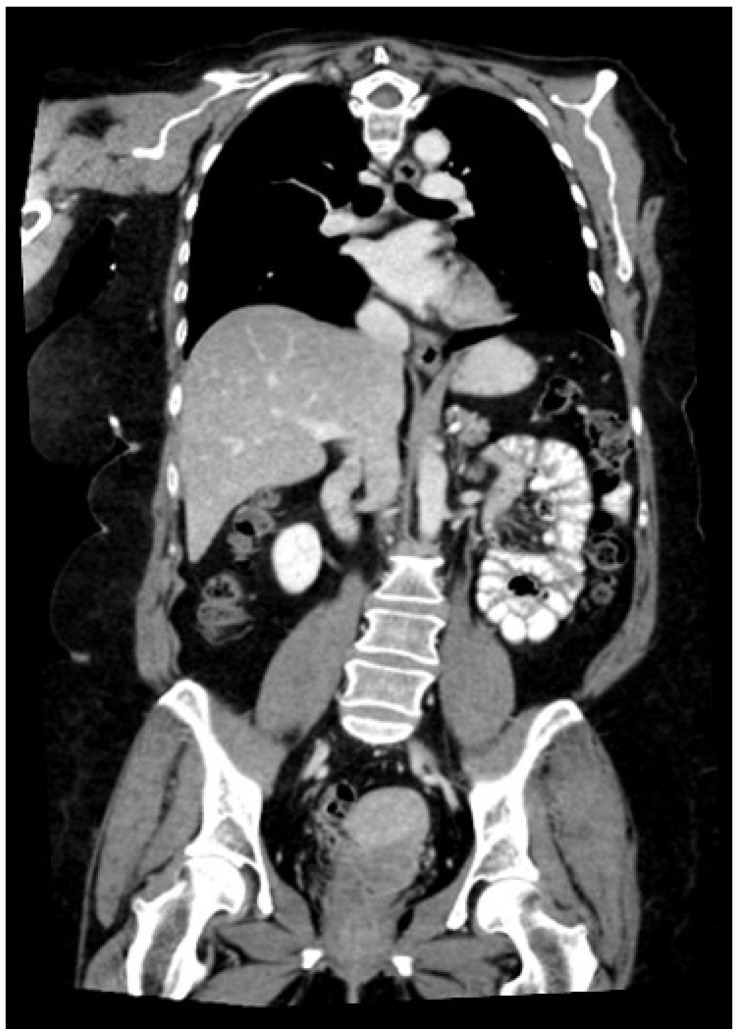
Contrast-enhanced CT of the chest, abdomen, and pelvis demonstrates no evidence of systemic malignancy.

**Figure 4:**
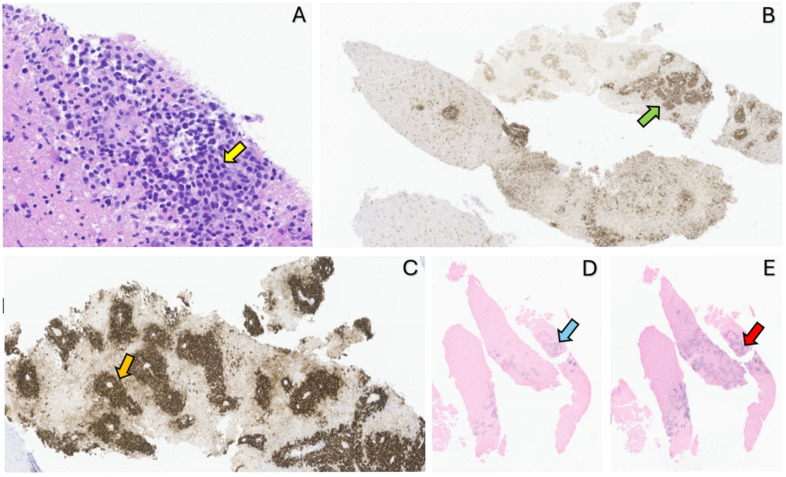
**Brain, right frontal lesion, biopsy.** (**A**) **(H&E, 40×):** Viable and necrotic brain tissue is infiltrated by multifocal, angiocentrically arranged clusters of atypical mononuclear cells exhibiting mitotic activity. Yellow arrow indicates atypical lymphoid cells. (**B**) **(BCL2 immunohistochemistry, 4×):** Tumor cells show positive reactivity for BCL2. Green arrow indicates representative BCL2-positive cells. (**C**) **(CD20 immunohistochemistry, 10×):** Tumor cells demonstrate strong diffuse CD20 positivity, confirming B-cell lineage. Orange arrow highlights angiocentric clustering around a vessel. (**D**) **(EBV-ISH, 4×):** EBV-encoded RNA in situ hybridization demonstrates positive staining within the atypical lymphoid infiltrate, supporting Epstein–Barr virus association. Blue arrow highlights EBV-positive tumor cells. (**E**) **(RNA EBV-ISH, 4×):** RNA EBV in situ hybridization shows stronger and more diffuse positivity within the atypical lymphoid population. Red arrow indicates representative areas of diffuse EBV-positive tumor cell staining.

## Data Availability

The raw data supporting the conclusions of this article will be made available by the authors on request.

## Abbreviations

AKI, acute kidney injury; BID, twice daily; CBC, complete blood count; CNS, central nervous system; CSF, cerebrospinal fluid; DLBCL, diffuse large B-cell lymphoma; EBV, Epstein–Barr virus; eGFR, estimated glomerular filtration rate; IV, intravenous; LDH, lactate dehydrogenase; MRI, magnetic resonance imaging; OD, once daily; PCNS-PTLD, primary central nervous system post-transplant lymphoproliferative disorder; PCR, polymerase chain reaction; PO, by mouth; PTLD, post-transplant lymphoproliferative disorder; SL, sublingual; SOT, solid organ transplant.
